# Fetal Supraventricular Tachycardia: What Do We Know up to This Day?

**DOI:** 10.3390/jpm15080341

**Published:** 2025-08-01

**Authors:** Sophia Tsokkou, Ioannis Konstantinidis, Vasileios Anastasiou, Alkis Matsas, Eleni Stamoula, Emmanuela Peteinidou, Antonia Sioga, Theodora Papamitsou, Antonios Ziakas, Vasileios Kamperidis

**Affiliations:** 1 1st Department of Cardiology, AHEPA Hospital, School of Medicine, Aristotle University of Thessaloniki, 54124 Thessaloniki, Greece; stsokkou@auth.gr (S.T.); vasianas44@gmail.com (V.A.); emma2405@windowslive.com (E.P.); aziakas@auth.gr (A.Z.); 2 Laboratory of Histology-Embryology, Department of Medicine, Faculty of Health Sciences, Aristotle University of Thessaloniki, 54124 Thessaloniki, Greece; ikonsc@auth.gr (I.K.); asioga@auth.gr (A.S.); thpapami@auth.gr (T.P.); 3 Laboratory of Experimental Surgery and Surgical Research ‘N.S. Christeas’, Medical School, National and Kapodistrian University of Athens, 11527 Athens, Greece; amatsas@med.uoa.gr; 4 Department of Clinical Pharmacology, School of Medicine, Aristotle University of Thessaloniki, 54124 Thessaloniki, Greece

**Keywords:** fetal tachyarrhythmias, fetal supraventricular tachycardia (SVT), fetal magnetocardiography (fMCG), digoxin, sotalol, flecainide, amiodarone, transplacental administration, direct fetal intramuscular injection, repeated intravascular injection

## Abstract

Fetal tachyarrhythmias, particularly supraventricular tachycardia (SVT) and atrial flutter (AFL), pose significant clinical challenges, especially when complicated by hydrops fetalis. This article provides a comprehensive review of the tachyarrhythmia types, the diagnostic modalities applied, and the therapeutic strategies followed in fetal tachyarrhythmias. Diagnostic techniques such as M-mode echocardiography and fetal magnetocardiography (fMCG) are highlighted for their capacity to provide real-time, high-quality assessments of fetal cardiac rhythms. The review, also, focuses on pharmacologic management via transplacental therapy, discussing the safety and efficacy of the key agents including digoxin, flecainide, and sotalol, under different clinical scenarios, such as hydropic fetus and renal impairment. In addition to transplacental administration, alternative approaches such as direct fetal intramuscular or intravascular injections are examined. These direct methods, while potentially more effective in refractory cases, carry risks that necessitate specialized expertise and careful consideration of maternal and fetal safety. The limitations of current evidence, largely based on small case studies and retrospective analyses, underscore the need for larger, prospective multicenter observational studies and randomized control trials to establish standardized protocols for fetal tachyarrhythmia management. Overall, this review advocates for a personalized, multidisciplinary approach, emphasizing early fetal tachyarrhythmias diagnosis, tailored treatment regimens that balances efficacy with safety, and rigorous monitoring to optimize outcomes for both the fetus and the mother.

## 1. Introduction

During gestational life the heart begins to exhibit detectable activity with the use of ultrasonography at approximately 5.5 to 6 weeks of gestation [1]. The normal fetal heart rate (FHR) varies depending on the gestational weeks and the stage of pregnancy. In early embryological development of the cardiovascular system, weeks 5 to 7, the FHR begins with slow conduction between 90 and 110 bpm, increasing to approximately 140 and 170 bpm by the 9th week and decreasing by 12th week to around 110 and 160 bpm during the third trimester, dropping slightly in the last ten gestational weeks [2,3,4,5]. Fetal arrhythmias are irregularities of the normal range of the cardiac rhythm, occurring in approximately 0.5 to 2% of all pregnancies [6,7,8]. The main types of them are supraventricular tachycardia (SVT) and atrial flutter (AFL) [8].

While most of these arrhythmias are of non-clinical significance, around 10% have the potential to compromise the state of cardiovascular compensation of the fetus [9]. Thus, a timely accurate diagnosis is of primary importance. Although cardiotocography and routine B-mode ultrasound provide initial surveillance by detecting sustained tachycardia and excluding gross structural anomalies, they cannot differentiate arrhythmia subtypes or elucidate underlying conduction mechanisms. Accordingly, precise prenatal diagnosis relies on advanced modalities, such as M-mode echocardiography, pulsed- and continuous-wave Doppler techniques, and, where available, noninvasive fetal magnetocardiography, to accurately define atrioventricular timing, circuit type, and ectopic activity. Specifically, in clinical practice, for the accurate assessment of the mechanical PR interval, a simultaneous mitral–aortic pulse-wave Doppler signal should be featured, and the interval from the onset of A-wave of the mitral inflow to the begging of the left ventricular ejection period (aortic pulse-wave Doppler) should be measured in milliseconds and compared to the normal fetal values [10].

There is a variety of treatment pharmacological choices for fetal tachyarrhythmias with reference to the type of drugs and the way of administration; digoxin, flecainide, sotalol, and amiodarone are the main options with differences in safety and efficacy for both the fetus and the mother, especially under specific circumstances such as hydrops fetalis [11]. Furthermore, although transplacental, fetal intracordal, fetal intraperitoneal, intra-amniotic, fetal intracardiac, direct fetal intramuscular injections, and oral maternal administration routes are all accepted, the quality of evidence supporting each varies, necessitating an individualized appraisal [12].

The aim of this narrative review is to provide a comprehensive synthesis of current evidence on fetal supraventricular tachyarrhythmias, with three objectives. First, to systematically classify the full spectrum of fetal tachyarrhythmias, emphasizing their underlying electrophysiological substrates and hemodynamic consequences. Second, to examine the strengths and limitations of available diagnostic modalities, in order to define a precise, mechanism-based diagnosis. Finally, to critically appraise current therapeutic options, outlining when and how to tailor each strategy to the individual fetal condition and maternal context. By integrating diagnostic precision with pharmacokinetic and safety considerations, this review proposes a personalized, multidisciplinary framework designed to restore fetal rhythm, reverse hydrops, and protect maternal health.

### Embryonic Conduction System of the Heart

The heart originates from an embryonic tissue called mesoderm approximately during the 18th to 19th day and starts beating and pumping blood at the 21st to 22nd day of gestation (3 weeks post fertilization) [13].

The embryonic conduction system of the heart is a specialized network of cells that develops early during cardiogenesis to regulate the rhythmic contractions of the heart. The formation of this system occurs as the heart transitions from a simple tubular structure to a more complex, chambered organ. The first signs of electrical activity are observed in the caudal region of the heart tube, which acts as the initial pacemaker. As development progresses, the sinoatrial (SA) node, atrioventricular node, and His–Purkinje system emerge as distinct components of the conduction system [14]. The SA node, which originates from the sinus venosus, becomes the primary pacemaker of the heart by the 5th gestational week [15]. The AV node and the bundle of His arise from the myocardium of the atrioventricular canal. These structures derive from specialized cardiomyocytes that exhibit slow or fast conduction properties, depending on their location and function. The Purkinje fibers, which form the terminal branches of the conduction system, develop from ventricular myocardium and facilitate rapid impulse propagation to ensure synchronized ventricular contraction. The development of the conduction system is influenced by molecular signals, including transcription factors such as TBX3 and NKX2-5, which regulate the differentiation and maturation of pacemaker and conduction cells [16,17]. Furthermore, genetic or structural perturbations, in the form of congenital heart defects, immune-mediated injury from maternal autoimmune diseases such as anti-Ro/SSA antibody positivity, hamartomatous infiltration in tuberous sclerosis complex, or myocardial remodeling in congenital and acquired (including infectious) cardiomyopathies, can disrupt these pathways, leading to maldevelopment of conduction tissue and the emergence of congenital arrhythmias or structural conduction defects.

## 2. Materials and Methods

The management of fetal supraventricular tachyarrhythmias is characterized by a sparsity of high-quality randomized trials, reliance on small observational series and case reports, and a wide spectrum of diagnostic modalities and therapeutic regimens. Faced with such heterogeneity in study designs, populations (hydropic versus non-hydropic fetuses), interventions (digoxin, flecainide, sotalol, amiodarone, transplacental versus direct fetal routes), and outcome definitions, a traditional systematic review would likely yield misleading pooled estimates and obscure critical nuances. By adopting a narrative synthesis framework, the full breadth of evidence could be captured, including rare case reports and series, without excluding valuable clinical insights simply because they do not conform to a narrow trial design, variations in diagnostic practices (M-mode echocardiography, fMCG) and treatment protocols could be mapped, highlighting real-world decision pathways and regional or institutional preferences methodological gaps could be identified, such as inconsistent reporting of time to cardioversion or long-term fetal outcomes, and thereby inform the design of future prospective multicenter studies.

A comprehensive literature search was performed to identify studies reporting on fetal supraventricular tachyarrhythmias, their diagnosis and management. PubMed, MEDLINE, Cochrane Library, and Embase electronic databases were queried from inception to 30 April 2025. Search terms combined controlled vocabulary and free-text words, and included the following keywords: “fetal tachyarrhythmia”, “supraventricular tachycardia” OR “SVT”, “atrial flutter” OR “AFL”, “hydrops fetalis”, “digoxin” OR “flecainide” OR “sotalol” OR “amiodarone”, “transplacental” OR “intramuscular” OR “intravascular”. References were reviewed for the identification of any additional relevant articles.

The review was restricted to full-text research studies published in peer-reviewed journals in the English language. Eligible studies had to meet the following criteria: original clinical studies of any type, including case reports, case series, prospective or retrospective cohorts, and randomized trials; the fetus must be diagnosed with supraventricular tachycardia or atrial flutter; detailed description of diagnostic modalities and pharmacological or invasive treatment regimens should be included; the studies must report outcomes on rhythm conversion, hydrops resolution, or adverse events. Additionally, due to scarce data on treatment and pharmaceutical regimens, it was decided to also include the results of any meta-analysis, if available on the subject. Duplicate records, review articles, protocols and guidelines, animal studies, conference abstracts and presentations, preprints, clinical trials under patient recruitment or without published results, ongoing clinical trials, and studies deemed irrelevant were excluded.

To ensure accuracy and objectivity, two independent reviewers (I.K. and S.T.) initially screened the titles and abstracts in a double-blinded process. For studies that passed this initial screening, the full texts were obtained and further evaluated to determine their final eligibility. Any discrepancies during the screening process were resolved by a third reviewer (T.P.).

## 3. Types of Fetal Tachycardia

Based on the Cincinnati Children’s Medical Center and Texas Children [18,19], there are four types of fetal tachycardias. Firstly, in sinus tachycardia (ST) the fetal heart rate is usually greater than 180 bpm but less than 200 bpm, with normal conduction. ST as recorded during fetal cardiac monitoring, can be transient—often reflective of normal fetal activity—or persist in a manner that is indicative of underlying pathology. Persistent ST has been correlated with several maternal and fetal comorbidities, including maternal hyperthyroidism, intrauterine infections, fetal anemia, and fetal hypoxia [18].

Supraventricular tachycardia (SVT) is recognized as the most common form of fetal tachyarrhythmia, and it is typically characterized by a heart rate exceeding 220 bpm originating from the atria [18,20].

Atrial flutter (AFL) is a macroreentrant atrial tachyarrhythmia in which the atria depolarize at rates of approximately 300–500 beats per minute, producing characteristic “saw-tooth” flutter waves on M-mode or Doppler, and are conducted to the ventricles in a fixed ratio (most commonly 2:1 or 3:1), yielding ventricular rates of 150–250 bpm. AFL is frequently associated with congenital heart disease or chromosomal abnormalities [21].

Lastly, ventricular tachycardia (VT) is the least common fetal tachyarrhythmia, defined by three or more consecutive ventricular contractions at rates typically exceeding 180–200 beats per minute, occurring independently of atrial activity and resulting in atrioventricular dissociation. In utero, VT is diagnosed by demonstrating a ventricular contraction rate that surpasses the atrial rate on M-mode or Doppler tracings. It has been described in association with myocardial inflammation (myocarditis), structural cardiomyopathies (congenital or acquired, including infectious forms), inherited channelopathies such as congenital long QT syndrome, and, less frequently, complete atrioventricular block [18].

While fetal supraventricular tachycardias (SVTs) encompass several mechanistic subtypes, the most common is atrioventricular re-entry tachycardia (AVRT), in which an accessory pathway conducts impulses between atria and ventricles in a circular fashion [8]. Less frequently, focal atrial tachycardia arises from an ectopic atrial focus, producing atrial rates of 200–300 bpm with variable AV conduction. Junctional ectopic tachycardia (JET) originates in or near the AV node and typically presents with AV dissociation or a VA/AV ratio > 1. On the other hand, AFL is a cavotricuspid isthmus-dependent macro-reentrant circuit, generating atrial rates between 300 and 500 bpm. On M-mode it produces a classic “saw-tooth” atrial waveform with 2:1 or 3:1 conduction and ventricular rates of 150–250 bpm [8].

Supraventricular tachyarrhythmias in the fetus are predominantly attributed to (SVT) and (AFL), which account for approximately 70–75% and 25–30% of cases, respectively [22,23]. Persistent arrhythmias elevate atrial and systemic venous pressures. The resultant hemodynamic burden of the high frequency tachycardia is driven by the increased ventricular myocardial stiffness and impaired diastolic filling, exacerbated by the loss of atrial contribution and sustained tachycardia which is poorly tolerated and may precipitate fetal congestive heart failure. Such diastolic cardiac failure can in turn lead to non-immune hydrops fetalis, placental edema, polyhydramnios, and, in severe cases, fetal demise. In addition to these fetal complications, significant maternal morbidities may also occur. Notably, mirror syndrome—a rare but life-threatening condition characterized by the development of maternal edema, hypertension, and proteinuria in the context of fetal hydrops—can manifest [24]. Importantly, with effective therapeutic intervention, resolution of fetal hydrops and reversal of mirror syndrome have been documented, underscoring the critical role of prompt diagnosis and management in these cases.

## 4. Diagnosis of Tachyarrhythmias

Accurate prenatal identification of fetal supraventricular tachycardia (SVT) and other tachyarrhythmias hinges on imaging the mechanical consequences of abnormal electrical activity. Although direct electrical recordings in utero remain impractical, a combination of ultrasound-based modalities and magnetocardiography provides detailed insight into atrial and ventricular timing, conduction patterns, and arrhythmia mechanisms.

Cardiotocography and routine B-mode sonography during pregnancy serve as initial surveillance tools. Cardiotocography flags persistently elevated baseline heart rates (>160 bpm) or reduced variability but cannot distinguish tachyarrhythmia subtypes. B-mode imaging confirms fetal viability and rules out structural lesions, guiding the need for advanced rhythm assessment [10].

M-mode remains the cornerstone for rhythm diagnosis, offering high temporal resolution of myocardial wall motion. This technique facilitates real-time visualization of myocardial motion and provides precise measurements of wall thickness, internal diameters, and most importantly heart rate, employing a single ultrasound beam to record the motion of intracardiac structures along a fixed line over time. By aligning that beam to intersect both atrial and ventricular walls, the examiner obtains sequential tracings of atrial (A-wave) and ventricular (V-wave) contractions. The temporal separation between those waves yields the mechanical PR interval, permitting differentiation of normal sinus tachycardia (atrial rate 160–200 bpm with 1:1 AV conduction), re-entrant SVT (ventricular rate 220–300 bpm with 1:1 conduction), and AFL (atrial rate 400–500 bpm with variable AV block). Owing to its high temporal resolution and straightforward image interpretation, M-Mode remains a first-line tool for rhythm analysis, though it provides no direct electrical data and is best applied when fetuses are in relatively stable positions without excessive motion [8,24,25,26].

Pulsed-wave Doppler (PWD) complements M-mode when acoustic windows are limited. Placed through the inflow and outflow tracts, for instance the mitral valve and left ventricular outflow, PWD yields atrial and ventricular Doppler waveforms, from which atrioventricular (AV) and ventriculoatrial (VA) intervals can be measured. Using Tissue Doppler Imaging by sampling myocardial tissue velocities at the atrial and ventricular walls, tissue Doppler allows direct timing of atrial contraction (A′ wave) and ventricular ejection (S′ wave). This technique offers higher temporal resolution than pulsed-wave Doppler but requires additional operator expertise. A consistent 1:1 A-to-V temporal relationship with rates over 200 bpm corroborates SVT; variations in this pattern suggest atrial flutter or other arrhythmias [8,27].

Moreover, the Superior Vena Cava–Ascending Aorta (SVC–AA) Doppler Flow Technique emerges as a powerful and practical tool for mechanistic diagnosis of fetal SVT. Simultaneous Doppler interrogation of the superior vena cava, as a marker of atrial contraction, and the ascending aorta, as a marker of ventricular ejection, yields a single, simultaneous tracing in which venous “a-waves” and arterial ejection spikes are unambiguously identified. Measurement of the interval from a-wave onset to ejection onset (the ventriculoatrial, or VA, interval) and from ejection onset back to the next a-wave (the atrioventricular, or AV, interval) permits precise quantification of atrioventricular relationships, even at fetal heart rates exceeding 200 bpm. Short VA intervals (<AV) with a prominent “cannon” a-wave identify re-entrant SVT through a fast-conducting accessory pathway and the recognition of this signature pattern can lead to prenatal conversion on first-line digoxin. Long VA intervals (>AV) distinguish atrial-automatic tachycardias (atrial ectopic foci or permanent junctional reciprocating tachycardia) and guided first-line sotalol therapy can achieve conversion. Finally, intra-atrial re-entrant tachycardias (flutter) can be confirmed by the presence of rapid a-waves (300–500 bpm) with fixed or variable AV block ratios, allowing targeted digoxin therapy and avoiding unnecessary multi-drug regimens. Additionally, measured intervals can also be converted into gestational age-adjusted z-scores using laboratory-derived normative data. A z-score ≤ –2 identifies a significantly short AV interval and correlates with prenatal pre-excitation and postnatal Wolff–Parkinson–White syndrome. Unlike M-mode imaging, which can lose atrial or ventricular motion markers in the presence of hydrops fetalis, maternal obesity, or unfavorable fetal lie, SVC/AA Doppler preserves clear flow-based landmarks regardless of myocardial contractility or image quality. This approach has demonstrated excellent intra- and inter-observer reproducibility and enables early differentiation of arrhythmia mechanisms, such as re-entrant SVT versus AFL, and also complements M-Mode through its ability to record both atrial and ventricular events continuously over several seconds, allowing simultaneous assessment of both chambers in one cardiac cycle, that enhances beat-to-beat reproducibility and facilitates recognition of intermittent conduction changes [8,26,27].

Furthermore, utilizing Continuous-Wave Doppler Echocardiography, a beat-to-beat measurement of isovolumetric contraction time (ICT), without reliance on intermittent sampling or angle-dependent pulsed-wave gates, can be recorded and, afterwards, Doppler envelopes are subjected to digital filtering to extract the interval between AV valve closure and semilunar valve opening over multiple consecutive beats. This quantitative analysis of that ICT then offers differentiation between compensated tachyarrhythmia, where ICT remains within the previously established normal range (~29 ± 2.5 ms), and decompensated states in which ICT is significantly prolonged (>35 ms) despite identical or even lower heart rates, objective monitoring of contractile reserve during serial examinations, such that a lengthening ICT reliably precedes or accompanies the onset of hydrops fetalis, and a non-invasive index of myocardial performance that is independent of fetal heart rate, thus providing a physiologic complement to conventional rate-based diagnosis of supraventricular tachycardia or atrial flutter [28].

In recent decades, direct recording of fetal cardiac electrical activity has become possible through non-invasive fetal electrocardiography (fECG) and fetal magnetocardiography (fMCG). fECG can enable precise arrhythmia classification and strives to capture fetal electrical potentials noninvasively through maternal abdominal electrodes. However, signal quality is often compromised by maternal and amniotic fluid interference, limiting its routine use. When adequately filtered, fECG can supplement ultrasound and fetal magnetocardiography (fMCG), particularly in late gestation when signal strength improves [29]. On the other hand, fMCG serves as an effective tool for recording the electrophysiological patterns associated with the initiation and termination of fetal reentrant supraventricular tachycardia [29]. It noninvasively records the magnetic fields generated by cardiac electrical currents. Unlike fECG, fMCG signals are minimally attenuated by the high resistance of fetal skin, yielding clear PR, QRS, and QT interval measurements, potentially enhancing characterization of complex arrhythmias, conduction delays, and repolarization abnormalities. fMCG delineates initiation and termination sequences of re-entrant SVT, measures precise conduction intervals, and can detect subtle atrial or ventricular ectopy that ultrasound alone may miss. However, they remain largely confined to specialized research centers due to requirements for noise-shielded environments, sophisticated hardware, and advanced signal-processing algorithms. Consequently, while fECG and fMCG represent the future of precise prenatal electrophysiology, routine clinical practice continues to rely on Doppler-based echocardiographic techniques [8,29].

Differential diagnosis of SVT from other tachyarrhythmias is critical for directing therapy. Atrial flutter is characterized by atrial rates of 300–600 bpm and typically 2:1 AV block, producing ventricular rates of 150–300 bpm with a sawtooth atrial pattern on advanced tracing. Ventricular tachycardia is rare in utero and manifests as dissociation of ventricular and atrial events, with ventricular contractions occurring independently of atrial timing. A fixed 1:1 AV relationship on M-mode or Doppler virtually excludes VT [24,25].

## 5. Treatment Options—Pharmaceutical Regimens

Effective pharmacologic management of fetal tachyarrhythmia requires careful consideration of both the route of administration and the specific pharmacokinetic properties of each medication. Different antiarrhythmic agents exhibit heterogeneity in fetal bioavailability and placental permeability, resulting in variable in utero efficacy. Moreover, these pharmacokinetic parameters change with advancing gestation. Specifically, during the third trimester, the expanded maternal plasma volume and increased placental perfusion can lead to hemodilution of transplacental drug concentrations, often necessitating an upward adjustment of the maternal dose to maintain therapeutic fetal levels. Additionally, possible fetal and maternal contraindications should be considered before drug administration to ensure safe SVT management [30].

### 5.1. Digoxin

Digoxin, a member of the digitalis glycoside class, is widely regarded as the first-line agent in treating fetal tachycardia due to its acceptable safety profile and proven therapeutic efficacy. During gestation, the physiological expansion of maternal blood volume and the concomitant increase in renal clearance significantly alter maternal pharmacokinetics [31]. Given digoxin’s high affinity for plasma protein, these changes necessitate the administration of larger and more frequent doses to ensure that adequate therapeutic concentrations are reached in the fetal circulation [32]. Digoxin demonstrates limited transplacental passage when the placenta is hydropic. In pregnancies complicated by fetal hydrops, the edematous state of the placenta impairs its ability to effectively transfer digoxin from the maternal to the fetal circulation. Consequently, digoxin is generally not favored as the first-line treatment in scenarios of decompensated heart failure, prompting clinicians to consider alternative therapies with improved placental permeability. However, digoxin can be routinely administered in combination with a second antiarrhythmic agent, most commonly flecainide, since it provides superior electrophysiological blockade, promotes restoration of sinus rhythm, and augments myocardial contractility due to its positive inotropic activity, thereby facilitating cardioversion, regression of hydrops, and reversal of congestive heart failure.

In terms of serum levels, although digoxin has a narrow therapeutic range of 1–2 ng/mL [30], it is recommended that higher digoxin levels of 2 and 2.5 ng/mL must be achieved during gestation due to the elevated glomerular filtration rate [30,32]. Digoxin levels are typically measured at least six hours after the initial dose to allow adequate distribution between serum and tissue compartments. Initiation of digoxin therapy for fetal tachyarrhythmia therefore requires in-hospital monitoring of both maternal and fetal parameters to optimize maternal tolerance and minimize the risk of toxicity until therapeutic plasma levels are achieved. Serial serum digoxin measurements and maternal electrocardiographic monitoring are integral to this approach, enabling timely dose adjustments to avert adverse effects in the mother and fetus.

Digoxin’s toxicity can clinically manifest as various cardiac arrhythmias, including atrioventricular blocks, atrial and ventricular fibrillation, bigeminy, and premature beats, necessitating regular assessment after drug administration. In terms of extracardiac manifestations, patients frequently experience gastrointestinal complaints (such as nausea, vomiting, and abdominal pain) and neurological symptoms (including confusion and generalized weakness). The risk of digoxin toxicity is significantly increased in the setting of renal dysfunction and electrolyte imbalances, specifically hypomagnesemia, hypercalcemia, and hypokalemia, which can alter the drug’s pharmacokinetics. Clinical judgment based on the patient’s signs and symptoms is of paramount importance in the timely detection of the toxicity, in addition to biochemical evidence and drug levels assessment [32,33].

### 5.2. Flecainide

Flecainide, a class Ic lipophilic antiarrhythmic agent, is characterized by comparatively low protein binding capacity, in contrast with digoxin. Flecainide achieves its antiarrhythmic properties by blocking sodium (Na^+^) channels, thereby inhibiting fast conduction within the myocardium. Its excellent maternal and fetal bioavailability, even in cases complicated by fetal hydrops, makes it an advantageous therapeutic option for the management of fetal SVT compared to digoxin. Clinical studies have established effective restoration of sinus rhythm in 43–85% of hydropic fetuses, with even higher success observed in non-hydropic fetuses. In the latter group, fetal flecainide levels approximate 90% of the maternal concentration, whereas in hydropic conditions, bioavailability is slightly reduced to around 80% of maternal levels. Given these favorable pharmacokinetic and pharmacodynamic characteristics, flecainide has been suggested as a first-line treatment option for hydropic fetuses suffering from SVT [30,33,34,35].

### 5.3. Sotalol

Sotalol hydrochloride is a non-cardioselective beta-adrenergic blocker, which has been used both as first- and second-line treatment in fetal SVT [36,37]. It acts by prolonging the duration of potentials in cardiac tissue and, thus, increases its refractory period, providing class III antiarrhythmic properties in addition to its beta-adrenergic blockage ability [38]. Sotalol is predominantly eliminated through renal excretion, meaning that its pharmacokinetics are heavily dependent on kidney function. Renal impairment can lead to accumulation of the drug, thereby increasing the risk of dose-dependent adverse effects, including potentially life-threatening arrhythmias such as torsade de pointes. Close monitoring of maternal renal function, serum electrolyte balance, and QTc are crucial to avoid proarrhythmia. As a non-cardioselective β-blocker, sotalol is contraindicated in patients with severe asthma or severe maternal obstructive pulmonary diseases. Its β-adrenergic blockade can produce maternal adverse events such as fatigue, dizziness, dyspnea, chest pain, palpitations, asthenia, bradycardia, nausea, and vomiting [36]. Its high bioavailability of about 90% does not appear to be significantly altered during pregnancy [37]. The suggested sotalol dose for treatment of fetal SVT ranges from 80 to 160 mg twice daily. To minimize the potential risk of maternal adverse events and/or intrauterine fetal death, a dosage regimen with an initiation dose of 80 mg of sotalol twice daily, a stepwise increase by 80 mg every three days, and a maximum daily dose of 480 mg or even higher given in three divided doses, has been suggested [37].

### 5.4. Amiodarone

Amiodarone, a class III antiarrhythmic agent, has poor transplacental transfer, with fetal–maternal concentration ratios ranging from 0.1 to 0.28, which is even more pronounced in the hydropic fetuses [30,39,40]. It is metabolized by the cytochrome P3A4 (CYP) pathway, which may be mostly upregulated in pregnancy. Amiodarone has the potential for a wide range of fetal and maternal adverse effects. Fetal or maternal hypo- or hyper-thyroidism have been reported. Neonates exposed to amiodarone during gestational life must have thyroid function studies performed due to the large proportion of iodine contained in each dose. Prolonged fetal exposure has also been linked with growth retardation and premature birth. Additionally, gastrointestinal symptoms, rash, pruritus, corneal microdeposits, peripheral neuropathy, myopathy, extrapyramidal tremor, cerebellar ataxia, insomnia, and nightmares are among potential maternal adverse effects [30]. The recommended oral starting dose is 800 to 1200 mg daily during 8 to 10 days and has a varying bioavailability of 30 to 80%. When the effect is achieved, the dose can be lowered to a maintenance dose of 400 to 800 mg daily [30,39]. Notably, there is limited experience with amiodarone for the treatment of fetal tachyarrhythmias, as it is only reported to be used in 7 of 537 cases in a systematic review of the treatment of fetal tachycardia [30]. In hydropic fetuses with supraventricular tachycardia refractory to digoxin, flecainide, or sotalol, maternal amiodarone is administered as a salvage therapy because its potent, broad-spectrum ion-channel blockade and positive inotropic properties can achieve rapid in utero cardioversion [30,37].

### 5.5. Arrhythmia-Specific First-Line Therapy

While fetal SVT and AFL share many hemodynamic sequelae, their underlying circuits differ in ways that markedly influence drug response. To begin with, AVRT or short VA interval SVT relies on dual-pathway reentry through an accessory AV connection. These tachycardias in non-hydropic fetuses usually respond to digoxin monotherapy. In hydropic or drug-resistant AVRT, flecainide is preferred because of superior placental transfer and more potent prolongation of accessory-pathway refractoriness. Moreover, atrial ectopic tachycardia (AET) and permanent junctional reciprocating tachycardia (PJRT or long VA interval SVT) are automatic or slowly conducting re-entry arrhythmias that are relatively digoxin-resistant. Sotalol, by lengthening atrial action potentials and producing secondary AV node blockade, is generally chosen as first-line therapy for these long-VA forms of SVT, with digoxin added only if rate control is inadequate. Furthermore, in non-hydropic AFL, which is a macro-reentrant tachycardia of the right atrium, typically 2:1–4:1 AV conduction, both digoxin and sotalol have high success rates, but sotalol’s class III properties shorten atrial refractoriness more potently and may be preferred when rapid cardioversion is required, especially in the setting of myocardial dysfunction. Finally, junctional ectopic tachycardia (JET) and VT are rare in utero. When recognized, amiodarone, with or without simultaneously administered digoxin, provides the most reliable direct suppression of automatic junctional foci or ventricular ectopy, albeit at the cost of higher maternal and fetal monitoring requirements. Table 1 maps each fetal tachycardia mechanism to its preferred first-line agent, together with the key rationale for that choice.

## 6. Treatment Options—Route of Drug Administration

### 6.1. Transplacental Route

When administering drugs for fetal SVT treatment, it is crucial to take into consideration both the method of delivery and their pharmacokinetic properties. The treatment of fetal arrhythmias often depends on the transplacental transfer of antiarrhythmic drugs administered to the mother, either orally or intravenously, which is the most widely applied route of drug administration. Most medications are transported across the placenta through simple diffusion. The rate of diffusion is influenced by several factors, including the concentration gradient between the mother and fetus, the drug’s lipid solubility, its binding to proteins, its molecular weight, and its level of ionization [30]. However, it should be noted that transplacental therapy is dependent on various factors which can lead to incomplete transfer of agents across the placenta. This can be attributed to the combination of shifting maternal circulating volumes throughout each trimester, increased maternal drug metabolism, increased maternal renal clearance as gestational age progresses to term, and potential placental edema; thus, medication doses must be adjusted accordingly [39].

#### 6.1.1. Pharmacological Evidence for Transplacental Route

Evidence in regard to efficacy and safety of transplacental antiarrhythmic drug administration stems from limited observational data.

A metanalysis conducted by Alsaied T. et al. regarding transplacental treatment for fetal tachyarrhythmia concluded that flecainide was superior to digoxin in terminating fetal SVT irrespective of the presence of hydrops fetalis. Specifically, digoxin achieved a lower rate of SVT termination compared with flecainide (odds ratio [OR]: 0.773; 95% confidence interval [CI], 0.605–0.987; I^2^ = 34%) [13], while there was no difference between digoxin and sotalol (OR: 1.009; 95% CI, 0.515–1.976; I^2^ = 68%) or between flecainide and sotalol (OR: 1.451; 95% CI, 0.996– 2.114; I^2^ = 0%). In subgroup analysis of fetuses with hydrops fetalis, digoxin had also lower rates of tachycardia termination compared with flecainide (OR: 0.412; 95% CI, 0.268–0.632; I^2^ = 0%) [41].

Similar findings were reported by the case of Takatsuka H. et al. who focused on the management of fetal SVT complicated by hydrops fetalis through transplacental therapy with digoxin in combination with flecainide. Authors report reduced efficacy of digoxin administered transplacentally due to impaired drug transfer. On the other hand, flecainide demonstrated better placental transfer rates, achieving higher concentrations in fetal plasma. Importantly, it was observed that the use of digoxin alone was insufficient in hydropic fetuses, but when it was combined with flecainide, it provided a better control of fetal heart rate [42].

Miyoshi T. et al. [43] conducted a multicenter study, involving a total of 49 patients enrolling from 15 medical institutions in Japan between the years of 2010 and 2017. Digoxin, sotalol, and flecainide were examined via transplacental administration and assessed for efficacy primarily by evaluating the resolution of fetal SVTs defined as the return to a normal sinus rhythm or a mean heart rate of less than 180 beats per minute using a fetal heart rate monitoring (40 min session to determine the heart rate and echocardiography; 30 min session to observe and confirm cardiac rhythm normalization). Digoxin was widely used due to its safety profile and clinician’s familiarity, and achieved a resolution rate of approximately 54.8% in cases without hydrops. Sotalol, introduced as a second-line therapy, demonstrated excellent efficacy, particularly for fetuses with SVT with long VA intervals, where it successfully resolved all SVTs [43]. Flecainide, a third-line option, was employed in resistant cases and showed strong efficacy, particularly in combination with other drugs. Overall, the study reported a high success rate of 89.8% in resolving fetal SVTs, although efficiency varied slightly based on the type of arrhythmia and presence of hydrops. Among others, these findings underscore the utility of combining antiarrhythmic medications in refractory cases, while emphasizing the importance of tailoring treatment protocols to individual fetal conditions [43]. In terms of adverse effects of transplacental treatment, these were observed in both mothers and fetuses. Maternal adverse events occurred in 78% of cases, though most were mild, such as nausea, vomiting, and non-clinically relevant bradycardia, and resolved with dose adjustments. Serious maternal adverse effects were uncommon, with only a single instance of Mobitz type II atrioventricular block occurring during combined digoxin and sotalol therapy. This conduction disturbance proved transient and resolved immediately upon temporary discontinuation of both agents. In fetuses, adverse events were observed in 12 of 50 cases (24%), including two deaths (4.1%). Both losses occurred in the context of refractory hydrops fetalis and decompensated supraventricular tachyarrhythmia—one case of atrial flutter and one of short VA SVT—despite temporary rhythm control, leading to hemodynamic sequelae incompetence, and were attributed to progressive heart failure rather than direct drug toxicity. No new complex ventricular arrhythmias or high-degree atrioventricular blocks emerged during therapy, and there were no reports of proarrhythmic events associated with the antiarrhythmic agents. Thus, in this cohort, fetal demise should be regarded as a consequence of severe, treatment-resistant tachyarrhythmia and not as a complication of the transplacental pharmacotherapy. In this multicenter cohort [43], five of forty-seven live-born infants (10.6%) developed treatment-related neonatal adverse events, of which the only serious complications were an episode of intestinal ileus, requiring temporary nasogastric decompression and parenteral support, and profound hypoglycemia (serum glucose 31 mg/dL) that was promptly corrected with intravenous dextrose infusion [43]. No new ventricular arrhythmias, conduction blocks, or proarrhythmic events were observed postnatally [43]. These findings reinforce that, although protocol-driven transplacental therapy achieves high rates of fetal cardioversion, meticulous neonatal monitoring, including serial abdominal examinations, blood glucose surveillance, and electrocardiographic assessment, is essential to detect and manage uncommon but potentially serious sequelae of in utero antiarrhythmic exposure [43].

#### 6.1.2. Practical Regimens and Monitoring

In pregnancy, drug pharmacokinetics are altered by increased maternal blood volume, cardiac output, and renal clearance. Consequently, both loading and maintenance doses often exceed nonpregnant regimens, and target maternal plasma or ECG end-points must guide therapy. Table 2 summarizes the loading schedules, therapeutic targets, maintenance dosing, and key safety monitoring steps for the four most commonly used transplacental agents in fetal SVT and AFL.

### 6.2. Non-Transplacental Route

Multiple alternative administration routes have been reported for direct fetal treatment including intracordal, intraperitoneal, intra-amniotic, intracardiac, and intramuscular injections (in the thigh of the fetus). Intraperitoneal, intra-amniotic, and intramuscular drug administration theoretically provide a sustained release of medication, but this may be limited by unpredictable drug delivery. Intracordal injection is effective but may require multiple injections, which increases the risk of procedural complication. Direct fetal therapy also involves the risk of the drug entering the maternal circulation and should be reserved for highly experienced centers [12]. Concerning direct fetal treatment, the mechanism involves bypassing the maternal circulation via intramuscular buttock, thigh, or intracordal routes, aiming to restore sinus rhythm successfully. While it may be effective in cases of severe hydrops and incessant SVT, it has also been associated with fetal toxicity, bradycardia, and even fetal cardiac arrest [12].

#### 6.2.1. Pharmacological Evidence for Direct Fetal Intramuscular Injection

Evidence regarding direct fetal intramuscular antiarrhythmic drug administration is scarce and limited to case reports and small observational studies [44,45]. Munoz JL et al. [44] discussed a case of refractory fetal SVT complicated by hydrops fetalis where the innovative use of direct fetal intramuscular injection of digoxin was applied. While traditional transplacental therapies like oral digoxin are the first line of treatment for fetal SVT, their efficacy significantly diminishes in the presence of hydrops, due to impaired placental drug transfer and cardiac decompensation with reports of failed cardioversion. Munoz JL et al. demonstrated that intramuscular digoxin achieved fast therapeutic effects in the fetus, offering an alternative option when fetuses are resistant to the conventional transplacental approach. However, it should be noted that, despite the successful cardioversion to sinus rhythm, fetal hydrops persisted, complicating the pregnancy further, resulting in maternal mirror syndrome, necessitating immediate delivery [44]. This could be due to delayed SVT treatment and prolonged fetal exposure to the tachyarrhythmia which could have led to refractory decompensation. The case report illustrates the potential benefit of invasive fetal therapy particularly for cases complicated by hydrops, although adequate evidence and validation of standardized protocols are currently lacking. Additionally, it emphasizes the importance of personalized approaches, considering gestational age, fetal condition, and maternal well-being in the decision-making process [45].

The use of direct fetal intramuscular injection of digoxin is further investigated by the study of Parilla BV et al. [45]. Maternally administered digoxin has long been regarded as the first-line treatment for fetal SVT, although its efficacy can be reduced in hydropic fetuses due to poor transplacental drug transfer. The impaired transfer to the fetus can be attributed to fluid accumulation and pathophysiological changes associated with hydrops, leading to subtherapeutic fetal serum drug levels and reduced responsiveness to the digoxin therapy. Parilla BV et al. [45] examined the use of combined maternal intravenous digoxin and fetal intramuscular digoxin therapy to overcome this limitation. The addition of direct fetal intramuscular digoxin therapy significantly shortened the time to initial cardioversion and sustained sinus rhythm when compared to maternal intravenous therapy alone. Specifically, for the eight fetuses treated initially with combined fetal intramuscular (FIM) and maternal intravenous administration (MIV) therapy, the mean time to restoring sinus rhythm was markedly reduced. These findings underscore the rapid and effective therapeutic potential of FIM administration in critical cases of refractory SVT. Furthermore, the study demonstrated that earlier resolution of hydrops fetalis was achieved with combined FIM and MIV therapy compared to MIV therapy alone (25 ± 21 days vs. 41 ± 37 days, respectively). By shortening the duration of hydrops, this combined approach could potentially reduce the risk of maternal complications such as mirror syndrome and improve overall maternal–fetal outcomes [45]. The above findings advocate a potential benefit of integrating FIM therapy into the standard transplacental treatment protocols for hydropic fetuses with SVT, particularly in refractory cases where transplacental therapy proves inadequate [45].

#### 6.2.2. Pharmacological Evidence for Repeat Intravascular Injection

Evidence regarding alternative non-transplacental drug administration such as intravascular injections is even more scarce. A case report of Mangione R et al. [46] describes the successful use of repeated intravascular injections of amiodarone directly into the umbilical vein for treating refractory fetal SVT complicated by hydrops, after failure of conventional transplacental therapies with digoxin and flecainide. The combination of direct amiodarone administration and evacuation of fetal ascites led to restoration of sinus rhythm, normalization of cardiac function, regression of hydrops, and ultimately successful labor. While this approach proved effective, this report emphasizes that direct intravascular fetal therapy should be reserved for severe, treatment-resistant cases due to significant associated risks including vein puncture. This method demonstrates a potential lifesaving option for managing persistent fetal arrhythmias when standard treatments are inadequate [46]. In Table 3 the efficacy and safety rates of antiarrhythmic agents for fetal SVT are summarized and in Table 4 the maternal and fetal adverse effects associated with antiarrhythmic drugs are highlighted.

## 7. Recommendations for Future Studies

This comprehensive review highlights current practices in the management of fetal SVT while highlighting the gap of evidence in the field. Although transplacental route for drug administration is considered the mainstay, it was shown to be ineffective in severe cases due to impaired drug transfer caused by congestive heart failure and generalized hydrops, which reduced fetal-to-maternal drug ratios. Also, the risks associated with direct fetal therapy, such as complications from umbilical vein puncture and drug injection, pose significant challenges and dilemmas. The presence of scarce literature, along with restricted exploration of alternative administration routes like intraperitoneal or intramuscular injections, underscores the need for further research and experience in the field.

**Figure 1:**
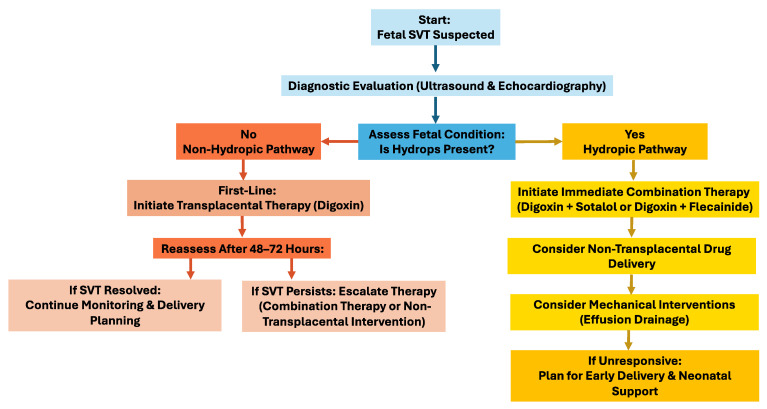
A Comprehensive algorithm for fetal supraventricular tachycardia (SVT) management with and without hydrops considerations.

It is important that future studies try to establish standardized algorithms for fetal SVT treatment, including direct therapy. Researchers and physicians should further explore alternative drug administration routes and the impact of effusion evacuation on cardiac function. Enhanced fetal monitoring techniques to evaluate drug transfer and efficacy in cases of hydrops would be beneficial. Collaboration across cardiology and obstetrics specialties can optimize outcomes, ensuring effective management while minimizing risks to the fetus and mother (Figure 1).

## 8. Conclusions

Management of fetal SVTs can pose significant diagnostic and treatment challenges. The mainstay for the management of these conditions is transplacental pharmacotherapy, with agents such as digoxin, flecainide, and sotalol forming the backbone of treatment. Although digoxin remains a first-line therapy, its efficacy is markedly diminished in hydropic fetuses due to impaired placental drug transfer. In contrast, flecainide establishes superior placental permeability and a higher success rate in restoring sinus rhythm, particularly in the setting of hydrops, while sotalol offers a feasible alternative with its combined beta-blocking and class III antiarrhythmic properties. However, none of these medications are risk-free and the potential adverse effects both for the mother and the fetus should be balanced. When transplacental therapy fails, especially in hydropic fetuses with evidence of decompensated heart failure, the preferred pharmacologic approach is a combination of flecainide and digoxin. Flecainide offers potent antiarrhythmic efficacy and reliably facilitates cardioversion, while digoxin contributes both to rate control and a positive inotropic effect that enhances biventricular performance. Indeed, if this dual-agent strategy proves insufficient, invasive fetal interventions, such as direct intramuscular administration of digoxin or repeated intravascular injections of amiodarone, may be employed, albeit only in specialized centers equipped to manage the procedural and hemodynamic risks inherent to these techniques.

Future research should aim to refine fetal monitoring techniques, establish standardized treatment protocols, and promote interdisciplinary collaboration between cardiology and obstetrics to enhance therapeutic outcomes and minimize adverse outcomes for both the fetus and mother.

## Figures and Tables

**Table 1:** Arrhythmia-specific first-line therapy.

Arrhythmia	Electrophysiology	First-Line Agent	Rationale
AVRT	Accessory-pathway reentry	Digoxin (non-hydropic)	AV node slowing prolongs re-entrant circuit cycle length; proven safety in non-hydropic SVT.
	short VA interval	Flecainide (hydropic/resistant)	Superior placental transfer; stronger accessory-pathway blockade; effective in hydropic AVRT.
AET	Atrial automatic focus	Sotalol	Class III prolongation of atrial refractoriness; rate control at AV node.
PJRT	Slow-conducting reentry	Sotalol	Lengthens both AV and VA limbs; effective in PJRT.
AFL	Macro-reentry in right atrium	Sotalol or Digoxin	Sotalol often faster conversion via atrial refractoriness; digoxin has modest efficacy in non-hydropic AFL.
JET	Junctional automatic focus	Amiodarone	Class III and multi-channel blockade suppresses automaticity; reserved for drug-resistant or decompensated cases.
VT	Ventricular automatic focus	Amiodarone	Potent suppression of ventricular ectopy; careful maternal–fetal monitoring required.

Abbreviations: AVRT = AV re-entrant tachycardia; AET = Atrial ectopic tachycardia; PJRT = Permanent junctional reciprocating tachycardia; AFL = Atrial flutter; JET = Junctional ectopic tachycardia; VT = Ventricular tachycardia.

**Table 2:** Transplacental anti-arrhythmic therapy: maternal dosing, targets, and monitoring [30,37].

Agent	Maternal Loading Regimen	Therapeutic Target	Maintenance Regimen	Key Maternal Monitoring
Digoxin	1 mg IV total over 24 h (0.5 mg IV q12 h × 2) [30] 2 mg PO total over 24–48 h (0.5–1 mg q6–12 h) [37]	Serum 2.0–2.5 ng/mL (6–8 h post-load) [30]	0.125–0.25 mg PO once daily (or 0.0625–0.125 mg PO BID) [30] 0.25 mg PO BID, adjust per levels [37]	ECG (PR interval), electrolytes (K^+^, Mg^2+^, Ca^2+^) at baseline and during dose adjustments [30,37] Serum digoxin level after load and at steady state; correlate clinically (toxicity risk > 2 ng/mL)
Flecainide	100 mg PO q8 h × 3 doses (300 mg total) [30] 100 mg PO q8 h (first-line) with maternal loading protocols varying from 100 to 200 mg/dose [37]	Trough 200–1000 ng/mL [30]	100 mg PO q8 h (max 400 mg/d) [30]	ECG (QRS duration) at baseline and regularly during therapy [30,37] Flecainide levels 6–12 h post-dose if dose ↑ or ECG changes [30] Monitor for CNS (dizziness, blurred vision) and GI (nausea) side effects
Sotalol	80 mg PO q12 h × 2 doses (160 mg total) [30] 80 mg PO TID, ↑ by 80 mg every 3 d (max 480 mg/d) [37]	QTc < 500 ms HR > 50 bpm Serum level < 2.5 mg/L [30]	80–160 mg PO q12 h (max 480 mg/d) [30]	ECG (QTc, HR) at baseline, during initiation, and after dose changes [30,37] Electrolytes, blood pressure; pulmonary status
Amiodarone	800–1200 mg PO daily (divided) for 7–10 d, then taper to maintenance [30] 1.8–2.4 g PO daily for 2–7 d, then 800 mg daily for 7 d, taper to 200–400 mg/d [37]	Monitor QTc, TFTs, LFTs [30]	400 mg PO once daily (divided) [30]	ECG (QTc), TFTs, LFTs at baseline and periodically during and after loading; chest radiograph if lung concerns [30,37]

Abbreviations: BID = twice daily; ECG = electrocardiogram; GI = gastrointestinal; HR = heart rate; h = hours; LFTs = liver function tests; PO = oral; QTc = corrected QT interval; TFTs = thyroid function tests; TID = three times daily; IV = intravenous; q = every; d = day; ↑ = increase.

**Table 3:** Efficacy and safety rates of antiarrhythmic agents for fetal SVT [8,22,23,24,25,26,27,28,29].

Drug	Efficacy in Non-Hydropic Fetuses	Efficacy in Hydropic Fetuses	Additional Notes
Digoxin	~55–70%	~30–40%	First-line transplacental therapy; however, its efficacy is reduced in hydropic fetuses due to impaired placental drug transfer. Meta-analysis data show lower termination rates compared with alternative agents in hydrops (OR ≈ 0.41 vs. flecainide).
Flecainide	~80–90%	~70–80%	Used as a second-/third-line option; displays superior placental transfer rates (fetal levels approximating ~90% of maternal concentrations in non-hydropic conditions) and appears especially effective in hydropic fetuses when combined with digoxin.
Sotalol	~65–75% (with some series reporting 100% in long VA SVT)	~60–70%	An effective alternative, particularly in cases of SVT with long VA intervals, with generally favorable safety. In small series, sotalol achieved 100% success in selected populations, although overall rates are similar to other agents when used broadly.
Amiodarone	~50–60%	~50–60% (limited data available)	Typically reserved as rescue therapy in refractory cases due to its poorer transplacental transfer (fetal–maternal ratios of ~0.1–0.28) and higher risk profile. Its use is generally limited because of maternal and fetal safety concerns.

**Table 4:** Maternal and fetal adverse effects associated with antiarrhythmic drugs [22,26].

Drug	Maternal Adverse Effects	Fetal Adverse Effects
Digoxin	Gastrointestinal upset (nausea, vomiting, abdominal pain) Headache Rare visual disturbances	Risk of digitalis toxicity, which may manifest as arrhythmias if serum levels exceed the therapeutic range; potential conduction disturbances
Flecainide	Dizziness Blurred vision Proarrhythmic potential requiring maternal ECG monitoring	Fetal conduction disturbances and arrhythmia if drug levels become excessively high; careful dosing is needed to avoid toxicity
Sotalol	Bradycardia Hypotension Dizziness QT interval prolongation, with potential risk for maternal arrhythmias	Fetal bradycardia, QT prolongation, with associated risk of arrhythmia, potential electrolyte imbalances
Amiodarone	Thyroid dysfunction (hypo- or hyperthyroidism) Elevated liver enzymes Pulmonary toxicity Photosensitivity and skin discoloration	Fetal thyroid dysfunction (transient hypothyroidism), Growth restriction and low birth weight; effects are related to iodine exposure and poor placental transfer

## Data Availability

No new data were created or analyzed in this study.
